# Sleep quality among patients with chronic illness in Ethiopia: systematic review and meta-analysis

**DOI:** 10.3389/fpsyt.2024.1365463

**Published:** 2024-05-30

**Authors:** Afework Edmealem, Belachew Tegegne, Girma Alem, Haymanot Zeleke, Temesgen Ayenew, Setarg Ayenew, Ayenew Sisay Gebeyew, Bereket Tomiso, Abuneh Getahun, Tirusew Wondie, Tiliksew Liknaw

**Affiliations:** ^1^ Department of Nursing, College of Medicine and Health Science, Debre Markos University, Debre Markos, Ethiopia; ^2^ Department of Nursing, College of Medicine and Health Science, Injibara University, Injibara, Ethiopia; ^3^ Department of Health Informatics, College of Medicine and Health Science, Debre Markos University, Debre Markos, Ethiopia; ^4^ Department of Public Health, Tropical College of Medicine, Debre Markos, Ethiopia; ^5^ South Wollo Zone Health Department, Dessie, Ethiopia

**Keywords:** hypertension, diabetes, cancer, sleep quality, chronic illness, Ethiopia

## Abstract

**Background:**

Poor sleep quality impedes the progression of chronic illnesses, while chronic illnesses themselves are caused by poor sleep quality. Despite this fact, there is no research that has been conducted in Ethiopia that provides a thorough estimate of the self-reported sleep quality among patients with chronic illnesses. In order to present a complete picture of poor sleep quality among diabetes, hypertension, heart failure, cancer, HIV/AIDS and epilepsy patients, this systematic review and meta-analysis was carried out.

**Methods:**

Systematic review and meta-analysis was conducted to estimate the quality of sleep among patients with chronic illness in Ethiopia. The Preferred Reporting Items for Systematic Review and Meta Analysis standard was followed in the reporting of this systematic review and meta-analysis. An extensive exploration of digital repositories, including PubMed, EMBASE, Cochrane, Africa Journal of Online, Google Scholar, and an advanced Google search, was conducted to obtain published studies until December 1st, 2023 detailing poor sleep quality of patients with chronic illness. STATA version 17 commands were used to create the pooled estimate. The I^2^ test and Egger’s test, respectively, were used to identify the presence of heterogeneity and publication bias. To manage heterogeneity, a subgroup analysis and random effect model were used.

**Results:**

A total 21 articles with a total of 7393 participants were included in the final systematic review and meta-analysis. The pooled estimate of poor sleep quality among patients with chronic illness was 52% (95% of CI: 48%, 59%; I^2 =^ 97.26%). In subgroup analysis, the highest pooled estimate of poor sleep quality was observed in cancer patients 63% (95% CI: (95% CI: 45% - 80%). Regarding to data collection period, the highest pooled estimate of poor sleep quality was seen during spring 68% (95% CI: 42% - 94%).

**Conclusions:**

Patients with chronic illnesses in Ethiopia had a high pooled estimate of poor sleep quality. Patients with cancer had the highest pooled estimate of poor-quality sleep compared with other patients. Patients with chronic illnesses had trouble sleeping in the spring, according to this systematic review and meta-analysis. Therefore, attention and intervention should be given to enhance the quality of sleep for patients with chronic illnesses.

## Introduction

Sleep is one of the most important aspects of life, as one-third of our lives are dedicated to sleep (1). It is vital to everyone’s life because it helps people unwind, revitalize their bodies, minds, and emotions, mend their physical bodies to enhance and preserve overall health, solidify their learning and memory, and refuel psychologically to preserve emotional equilibrium and well-being (1–3). The importance of maintaining adequate sleep quality and quantity has relevance beyond reducing healthcare spending, improving morale, and improving productivity (4). In terms of preserving general health, sleep is just as crucial as diet or exercise (3). Poor sleep quality has been linked to a lower chance of living a longer life (5), along with other risk factors such obesity and weight gain, high blood pressure, high cholesterol, diabetes, atherosclerosis, poor mental health, alcoholism, smoking, bad eating habits, and sedentary behavior (6–8). Additionally, sleep plays a significant role in brain activities that are critical for work, such as decision-making, attentiveness, and drowsiness (9).

Chronic illness patients face several challenges. The primary complaint among them is sleep disorders (10). Sleep problems included inadequate sleep duration, insomnia, snoring, poor sleep quality, obstructive sleep apnea and restless legs syndrome (1, 11). Patients with chronic illnesses have been shown in studies to go years without getting enough sleep (12) which impaired their quality of life (13). Poor sleep quality impedes the progression of chronic illnesses, while chronic illnesses themselves are caused by poor sleep quality (14). For instance, a comprehensive review found that individuals living with HIV/AIDS are at risk to develop poor sleep quality (15). The hypothalamic-pituitary-adrenal axis, disturbed circadian rhythms, proinflammatory reactions, elevated sympathetic nervous system activity, and metabolic impacts are some of the mechanisms behind the multifaceted relationship between poor sleep quality and chronic illness (2, 16).

Poor sleep quality has become an increasing public health problem in modern society, and there is a high correlation between poor sleep quality and the development of late-chronic diseases (2). The magnitude of poor sleep quality is high among patients with chronic illnesses. A systematic review of 20 studies among patients with chronic kidney diseases showed that the prevalence of poor sleep quality was 11% to 97% (17). Another systematic review of 24 studies among hypertension patients in China revealed that poor sleep quality ranges from 14.9% to 85.7% (18). Furthermore, the prevalence of poor sleep quality among cancer patients ranges from 16% to 93% (19).

Poor sleep quality has a negative effect on self-rated health or one’s perception of his or her health (20), life expectancy (5), quality of life (13), impaired immune system, hampered physical performance, affected cell growth and repair, deteriorated neuronal connections and neuronal malfunctions (9), deleterious health consequences, including an increased risk of hypertension, diabetes, atherosclerosis, obesity, depression, heart attack, and stroke (8, 10), results in vehicle and fall accidents (9), high health care expenditure (10), absenteeism from the workplace, presenteeism/productivity loss (2, 10), and in general, it increases the disease burden (3). The impact of poor sleep quality on the patients’ quality of life is much in African population due to the rapid increment of aging population and chronic disease conditions.

Beyond reducing health care costs, sleep intervention has relevance to improving morale and productivity (21). Studying the prevalence of poor sleep quality at the national level warrants health care providers and policymakers to give more attention and prepare themselves for intervention. The quality of sleep among Ethiopian patients with cancer, HIV/AIDS, diabetes, heart failure, hypertension, and epilepsy is not consistent. Moreover, no research has been conducted in Ethiopia that provides a thorough estimate of the pooled prevalence of poor sleep quality among patients with chronic illnesses. In order to present a complete picture of poor sleep quality among Ethiopian patients with chronic illnesses, this systematic review and meta-analysis was carried out.

## Methods

### Study design and setting

Systematic review and meta-analysis of both published articles conducted in Ethiopia to estimate the quality of sleep among patients with chronic illness. Ethiopia is one the developing country in East part of Africa. It has 13 regions and 2 city administrations. The regions are Tigray, Afar, Amhara, Oromo, Ethiosomali, Benshangul gumz, Central Ethiopia, Sidama, South West Ethiopia, South Ethiopia, Gambela, and Hareri. The two city administrations are Addis Ababa and Dredawa (22).

### Eligibility criteria

#### Inclusion criteria

In this systematic review and meta-analysis, all published articles that were done in Ethiopia were included. All studies that reported the quality of sleep among patients with chronic illnesses such as diabetes, heart failure, hypertension, cancer, HIV/AIDS and epilepsy were included. All types of studies that were published in English and conducted among all stages of chronic illness and published until December 1st, 2023, were included.

#### Exclusion criteria

Studies that failed to report sleep quality based on the Pittsburgh Sleep Quality Index (PSQI) tool were excluded from this review. Studies that were conducted among patients who were taking sleep medication were excluded. Additionally, studies such as case reports, case studies, editorial letters, reports, and qualitative studies were excluded from the study.

### Data source and search strategy

Before going to the extensive searching, the presence of systematic review and meta-analysis protocol on sleep quality among patients with chronic illness at PROSPERO was checked. After that, both published articles and grey literatures that assessed sleep quality among patients with chronic illness in all regions of Ethiopia were used as sources of data. The review was conducted by using the Preferred Reporting Items for Systematic Review and Meta Analysis (PRISMA) guideline (23). The search strategy was developed using the Population (Patients with chronic illness), Intervention, Comparison (patients without chronic illness), and Outcome (sleep quality) searching guide. For published articles, an intensive search of online databases such as PubMed (MEDLINE), EMBASE, Cochrane, Africa Journal of Online, Google Scholar, and an advanced Google search was made. During systematic search, the word ((((((((“sleep quality”) OR “poor sleep quality”) AND patients) OR “chronic illness patients”) OR “patients with hypertension”) OR “patients with diabetes”) OR “patients with heart failure”) OR “patients with HIV/AIDS”) OR “patients with cancer”) OR “patients with epilepsy”) AND Ethiopia) was used. All articles in reference lists were searched to include additional studies and reports in the review and analysis. A systematic search of the literature was made from November 11, 2023, until December 1, 2023.

### Measure of outcome

There are various ways to quantify the quality of sleep. Nonetheless, the Pittsburg Sleep Quality Index (PSQI) was the most often utilized instrument. Its validity was assessed in Ethiopia among community-dwelling adults (24). The PSQI evaluates sleep quality by asking questions regarding, e.g., the respondent’s self-reported duration of sleep, sleep latency, and sleep arousals for the 1 month preceding the time of evaluation. The 19 questions that make up the PSQI are divided into seven categories. Each questionnaire response is assigned a number between 0 and 3, where 0 denotes no problems and 3 denotes serious problems. The sum of the response ratings from each of the seven question groupings makes up the final score. The total score (global score) can range from 0 to 21. The lower scores indicate better sleep quality, and the higher scores indicate poor sleep quality. Chronic illnesses are disease conditions that include diabetes, hypertension, heart failure, HIV/AIDS, cancer, and epilepsy.

### Quality assessment and critical appraisal

In this systematic review and meta-analysis, included studies were cross sectional studies. There is only one case control study. Hence, the quality of included studies were assessed by a 8 item critical appraisal tool adopted from Joanna Briggs Institute (JBI) (25) and considered high quality when the sum of JBI items is above 70%. This is a tool used for the evaluation of prevalence studies. AE and TA assessed the methodological quality of eligible articles independently. The difference in extraction was managed through discussion and the third author. All articles scored above half of the score were included in the systematic review and meta-analysis.

### Data extraction/abstraction

A Microsoft Excel spread sheet was used to generate the pre-piloted format in which AE, BT, SA, and AS extracted the data from the included literature. First author names, year of publication, region, sample size, population categories, study design, study area (institution vs. community), PSQI cut-off point, data collecting time, and prevalence of poor sleep quality were retrieved from each article. Following a conversation among the authors to resolve the disagreement, AE collated the data that had been extracted from the authors.

### Statistical analysis

The extracted data were exported from the Microsoft Excel spreadsheet and entered the STATA version 17 command window (26). Using the I-squared statistic, the presence of statistical heterogeneity within the included papers was evaluated prior to conducting the primary meta-analysis. I^2^ value ranges between 0 and 100% whereby I^2^ > =75% indicate high heterogeneity across the studies. Additionally, Egger’s tests and a funnel plot were used to determine whether publication bias existed. Following that, STATA meta set command was used to complete the pooled estimate. To control heterogeneity, a subgroup analysis of the included studies was conducted based on the categories of populations and the cutoff point. Additional advanced statistical analyses, such as meta-regression to identify the potential sources of heterogeneity and sensitivity analysis to investigate the influence of a single study on the overall pooled estimate, were performed. The trim and fill test were done to minimize publication bias. The findings of this study were presented using tables and forest plots with 95% confidence intervals (CI).

## Result

### Identification of studies

**Figure 1:**
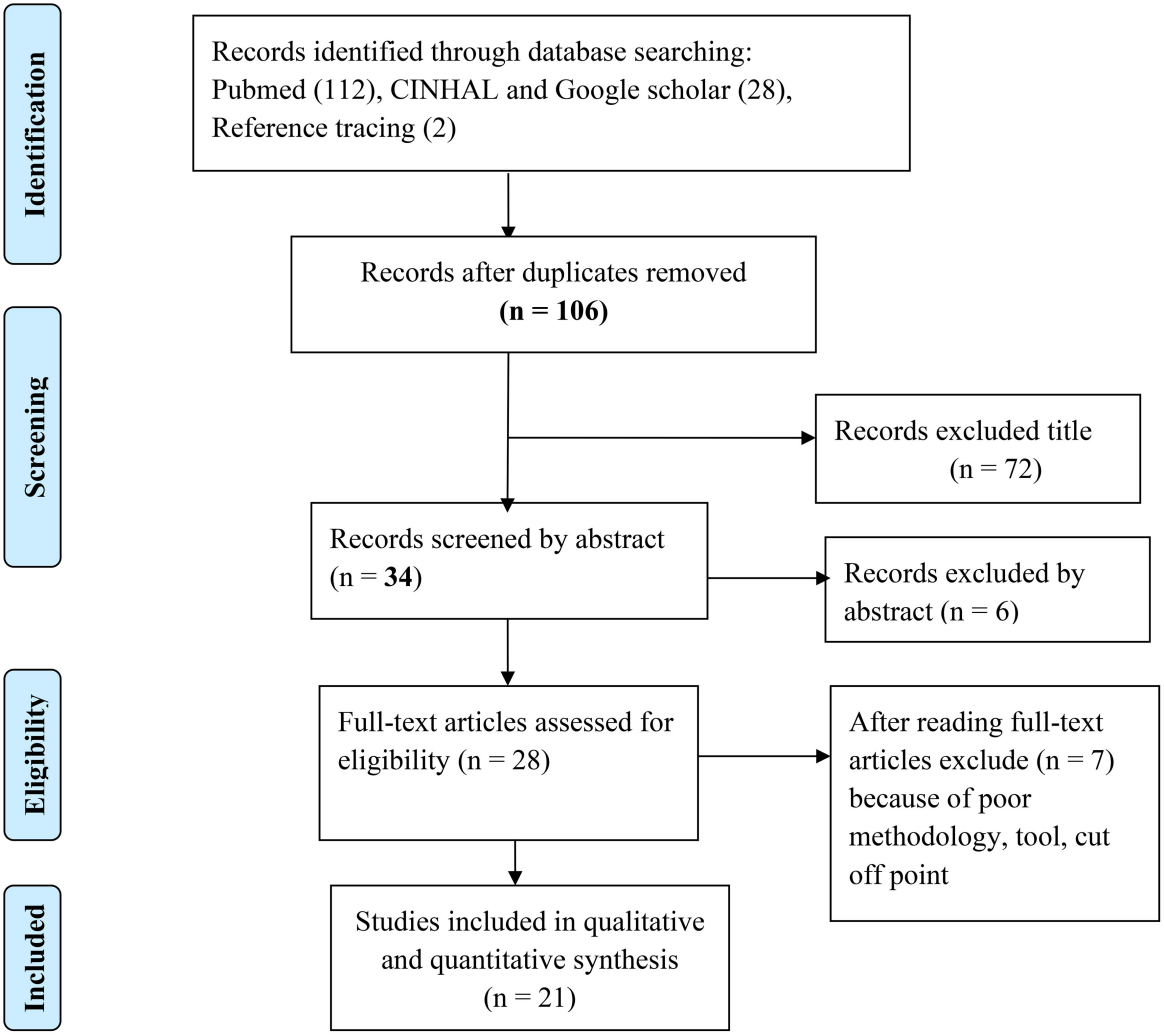
PRISMA flow diagram that shows study selection for meta-analysis of sleep quality among patients with chronic illness.

Using advanced searching, 142 articles were found in Pubmed, EMBASE, Google Scholar, and Google. Reference tracing was used to add two articles to the overall number of articles. One hundred and six papers were screened using their title and abstract after 36 duplicate articles were eliminated. A total of 28 articles were screened for their full text. Following that, a total of 21 articles that passed the eligibility requirements and quality assessment were included in the final systematic review and meta-analysis (**Figure 1**).

### Description of included studies

A total of 21 articles with 7393 study participants were included in the final systematic review and meta-analysis. All the articles were published from 2015 till 2023. All of the articles were done at institutional level. From the total included studies, 4 of them were done in Addis Ababa (27–30) and 11 studies were done in the Amhara region (29, 31–40). The other five articles were conducted in the Oromia region (41–45), and one article was done in the Sidama region (46). Regarding the study population, five studies were conducted on diabetes patients (29, 32–34, 41), and the other six studies were conducted on HIV/AIDS patients (29, 38, 39, 44, 46, 47). One of the articles used case control as a study design (32). Three articles used non-probability sampling, such as convenient sampling (32, 41, 42). Five of the articles reported poor sleep quality for those patients whose PSQI score was ‗ 5 (28, 37, 42, 43). The rest 16 studies used PSQI score >5 to declare the presence of poor sleep quality. The largest sample size was 565, which was obtained from a study conducted in the Amhara region among epileptic patients in 2021 (37). The data for three studies were collected during the winter season (34, 35, 38), and similarly the data for three studies were collected during the summer season (29, 32, 45) (**Table 1**).

**Table 1:** characteristics of included studies, 2023.

Author	Publication year	Region	Study area	Type of population	Sampling technique	Sample size	Study design	Data collection period	Cut off point	magnitude of sleep quality (%)
Abebe E. et al. (48)	2023	Addis Ababa	Institution	Cancer	Systematic	264	Cross sectional	Autumn	>5	53.8
Endeshaw D. et al. (31)	2022	Amhara	Institution	Cancer	Systematic	410	Cross sectional	Autumn	>5	71.5
Zewdu D. et al. (49)	2022	Amhara	Institution	Diabetes	Systematic	292	Cross sectional	Autumn	>5	50.7
Birhanu et al. (34)	2020	Amhara	Institution	Diabetes	Systematic	430	Cross sectional	Winter	>5	47.2
Edmealem A. et al. (36)	2020	Amhara	Institution	Diabetes, HF Hypertension	Systematic	384	Cross sectional	Autumn	>5	36.5
Edmealem A. et al. (35)	2021	Amhara	Institution	Diabetes, HF Hypertension	Systematic	344	Cross sectional	Winter	>5	36.0
Adem K. et al.(50)	2020	Addis Ababa	Institution	Epilepsy	Systematic	415	Cross sectional	Autumn	‗5	65.5
Simie T et al. (37)	2021	Amhara	Institution	Epilepsy	Systematic	565	Cross sectional	Autumn	‗5	68.8
Balcha. et al. (42)	2015	Oromia	Institution	Heart Failure	Convenient	278	Cross sectional	Spring	‗5	81.65
Getahun Y. et al. (43)	2021	Oromia	Institution	Heart Failure	Systematic	111	Cross sectional	Autumn	‗5	37.8
GebreEyesus et al. (51)	2023	Oromia	Institution	HIV/AIDS	Systematic	419	Cross sectional	Winter	>5	36.0
Bedaso A. et al. (46)	2020	Sidama	Institution	HIV/AIDS	Systematic	389	Cross sectional	Autumn	>5	57.6
Bedaso A. et al. (52)	2023	Addia Ababa	Institution	HIV/AIDS	Systematic	388	Cross sectional	Autumn	>5	73.7
Mengistu et al. (47)	2021	Addis Ababa	Institution	HIV/AIDS	Systematic	396	Cross sectional	Autumn	>5	55.6
Tesema E. et al. (53)	2020	Oromia	Institution	Hypertension	Systematic	279	Cross sectional	Summer	‗5	35.5
Jemere et al. (41)	2019	Oromia	Institution	Diabetes	Consecutive	198	Cross sectional	Autumn	>5	55.6
Mersha et al. (32)	2023	Amhara	Institution	Diabetes	consecutive	126	Case control	Summer	>5	45.2
Shibabaw et al. (29)	2023	Amhara	Institution	Diabetes	Systematic	407	Cross sectional	Summer	>5	33.9
Adane M. et al. (39)	2022	Amhara	Institution	HIV/AIDS	Systematic	399	Cross sectional	Spring	>5	55.1
Ayanaw T. et al. (40)	2022	Amhara	Institution	Hypertension	Systematic	563	Cross sectional	Autumn	>5	37.7
Abdu and Dule (44)	2020	Oromia	Institution	HIV/AIDS	Systematic	336	Cross sectional	Autumn	>5	57.1

### Heterogeneity test and publication bias

**Figure 2:**
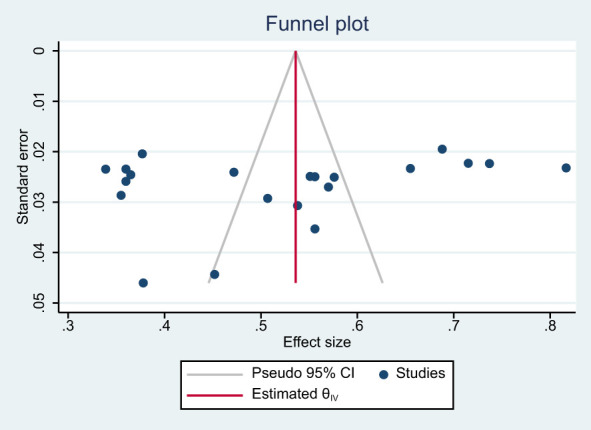
Funnel plot to see the presence of publication bias.

Before pooling the estimated effect, the presence of heterogeneity and publication bias were evaluated. Heterogeneity between included articles was assessed statistically by using I-squared statistic. According to the result, there was high heterogeneity between the included studies (I^2 =^ 97.26; P = 0.000). The presence of publication bias was statistically assessed by Egger’s weighted regression, Begg’s method and funnel plot. P value of Egger’s and Begg’s test was 0.209 and 0.349 respectively, which indicates that there is no publication bias. However, the funnel plot showed asymmetrical distribution of studies inside the funnel which implies that there is a publication bias (**Figure 2**).

### Sleep quality among patient with chronic illness in Ethiopia

**Figure 3:**
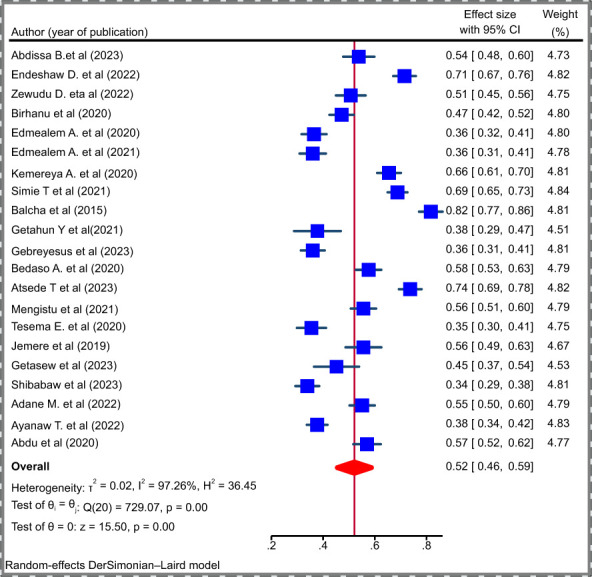
Forest plot depicting the pooled prevalence of poor sleep quality among patients with chronic illness, 2023.

After testing heterogeneity and the presence of publication bias, the effect size was pooled by using STATA version 17. Since there is considerable heterogeneity between the studies (I^2 =^ 97.26; P = 0.000), the main meta-analysis was performed using a random effect model. As it is shown in **Figure 3**, the pooled estimate of poor sleep quality for patients with chronic illness was 52% (95% CI: 46 - 59%) (**Figure 3**).

### Meta regression

Meta regression was performed to identify the sources of heterogeneity between the studies. In this systematic review and meta-analysis, meta regression was performed by type population, cut off point for PSQI score, data collection period and region to evaluate whether these variables are sources of heterogeneity between the studies. However, none of them were sources of heterogeneity, in which the P value was above 0.05 (**Table 2**).

**Table 2:** Meta regression of selected variables, 2023.

Heterogeneity sources	Coefficients	Std. error	P value
Type of population	-0.004	0.016	0.777
Study period	-0.040	0.029	0.168
Cut off point for PSQI	0.103	0.078	0.188
Region	-0.015	0.043	0.724

### Subgroup analysis for poor sleep quality

Subgroup analysis is done by type of population, season of data collection and cutoff point of PSQI to minimize heterogeneity between the included studies. As it is shown in **Table 3**, the highest pooled estimate poor sleep quality was seen among patients’ cancer patients 67% (95% CI: 64%-71%). The pooled estimate of poor sleep quality among diabetes patients was 36% (95% CI: 33% - 40%). Moreover, the pooled estimate of poor sleep quality among people living with HIV/AIDS was 56% (95% CI: 48 – 68%). The highest poor sleep quality was seen among studies whose data was collected during spring (68%: 95% (42% - 94%) (**Table 3**).

**Table 3:** Sub group analysis by type of population, cut off point and data collection period.

Subgroup	Category	Number of studies	Total sample size	Proportion of poor sleep quality (95% CI)	I^2^	P value
Type of population	Cancer patients	2	674	63 (45–80)	95.41%	0.00
	Diabetes patients	5	1453	48 (39–54)	88.89%	0.00
	Diabetes, hypertension and heart failure	2	728	36 (33–40)	NA	NA
	Epileptic patients	2	980	67 (64–71)	15.11%	0.28
	Heart failure patients	2	389	NA		
	HIV/AIDS patients	6	2327	56 (48–68)	98.33%	0.00
	Hypertension patients	2	842	37 (34–40)	NA	
Cut off point	>5 of overall PSQI score	16	5745	50 (44–57)	96.39%	0.00
	‗5 of overall PSQI score	5	1648	58 (42–74)	97.96%	0.00
Data collection period	Autumn	13	4711	56 (48–63)	97.36%	0.00
	Winter	3	1193	40 (32–47)	85.94%	0.00
	Spring	2	677	68 (42–94)	98.36%	0.00
	Summer	3	812	37 (32–43)	61.05%	0.08

NA, Not applicable.

### Sensitivity analysis

**Figure 4:**
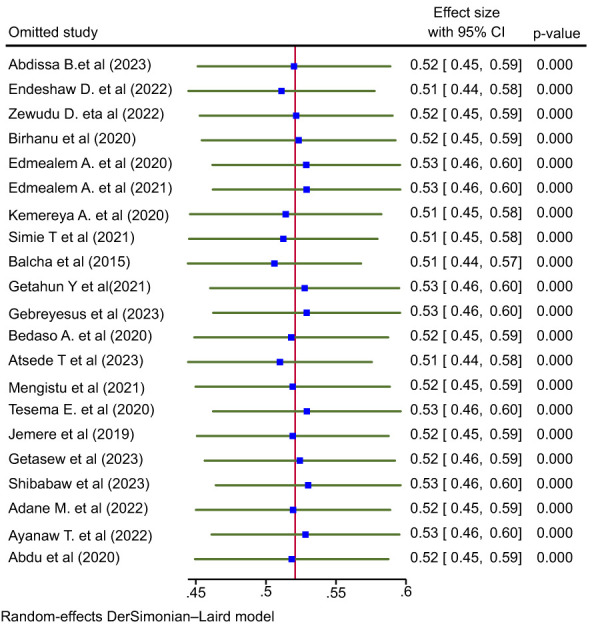
Leave - one- out meta- analysis to see the influence of the single study on the pooled effect size, 2023.

Sensitivity analysis was done to evaluate whether the pooled effect size was influenced by individual studies or not. As it is shown in **Figure 4**, there is no any study which influences the overall pooled estimate of poor sleep quality among patients with chronic illness. The pooled effect size after omitting the individual study was inside the confidence interval of the overall pooled effect size (all the effect sizes after omitting a single study were between 48% and 59%) (**Figure 4**).

## Discussion

The prevalence of poor sleep quality among patients with chronic illness in Ethiopia is not known. However, understanding the overall quality of sleep among patients with chronic illness is valuable for different stake holders including health care providers to tackle the adverse prognosis and decrease the burden of the diseases. This systematic review and meta-analysis revealed that the pooled estimate of poor sleep quality among patients with chronic illness in Ethiopia was 52% (95% CI: 48% -59%). The prevalence of sleep quality in this study when compared to other studies, it is in line with the finding of the pooled estimate of poor sleep quality among chronic kidney disease patients (59%) (17) and a single study in Nigeria among chronic kidney disease patients (54). Additionally, the pooled prevalence of poor sleep quality for patients with chronic illness in this study is comparable to the global prevalence of poor sleep quality among cancer patients(57.4%) (19), and the pooled prevalence of poor sleep quality among stroke patients (53%) (55). On the contrary, the finding of this review is higher than the pooled prevalence of poor sleep quality among patients with Inflammatory bowel disease (19). Moreover, the finding of this systematic review and meta-analysis is lower than the pooled prevalence poor sleep quality among HIV/AIDS patients (63%) (44). The possible justification for this discrepancy might be the difference in study population, eligibility criteria, the treatment modality and the cutoff point in the PSQI score. Most of the studies pooled the prevalence of poor sleep quality for a single type of population in their analysis unlike to this study which pooled the prevalence poor sleep quality for different types of chronic illnesses. The quality of sleep quality is different for different disease conditions.

According to the subgroup analysis, the highest pooled estimate of poor sleep quality was seen among cancer patients. This might be occurred due to the treatment effect and pain (56). According to a systematic review, the treatment given to cancer patients affects the sleep quality (57). The pooled estimate of poor sleep quality among cancer patients was 63% (95% CI: 45% - 80%). In this subgroup analysis, the lowest pooled estimate of poor sleep quality was observed among hypertension patients. Hypertension is a silent killer, and it has no any suffering symptoms in contrast to cancer, HIV/AIDS, diabetes, epilepsy and other chronic illnesses. As a result, there is no significant factor that hinder and interfere sleep quality of hypertension patients. The difference in socioeconomic status, pathogenies of the disease condition, environmental factors, treatment modality, study period and life style of patients might be the other justifications for the discrepancy between the highest and lowest pooled estimates.

Seasonal variations and environmental factors have an impact on the quality of sleep. This systematic review and meta-analysis found that studies using data gathered in the spring had the highest pooled estimate of poor sleep quality 68% (95% CI: 42% - 94%). The chilly weather in Ethiopia throughout the spring may be the cause of this. The pooled estimate of poor sleep quality was lowest among patients with chronic diseases who were interviewed in the summer 37% (95% CI: 32% - 43%). This could be because of the high temperatures that make Ethiopia’s summertime more comfortable.

## Limitation

This systematic review and meta-analysis has its own limitations. The presence of high level of heterogeneity among included studies is the main limitation. Included studies use different cut off point to estimate the prevalence of poor sleep quality.

## Conclusion

Patients with chronic illnesses in Ethiopia had a high pooled estimate of poor sleep quality. Patients with cancer had the highest pooled estimate of poor-quality sleep compared with other patients. Patients with chronic illnesses had trouble sleeping in the spring, according to this systematic review and meta-analysis. Therefore, healthcare professionals, the ministry of health, and other stakeholders should give attention and intervention to enhance the quality of sleep for patients with chronic illnesses.

## Data availability statement

The original contributions presented in the study are included in the article/supplementary material. Further inquiries can be directed to the corresponding author.

## Author contributions

AE: Conceptualization, Data curation, Formal analysis, Investigation, Methodology, Resources, Software, Supervision, Validation, Visualization, Writing – original draft, Writing – review & editing. BTe: Writing – original draft, Writing – review & editing. GA: Writing – original draft, Writing – review & editing. HZ: Writing – original draft, Writing – review & editing. TA: Writing – original draft, Writing – review & editing. SA: Writing – original draft, Writing – review & editing. AS: Writing – original draft, Writing – review & editing. BTo: Writing – original draft, Writing – review & editing. AG: Writing – original draft, Writing – review & editing. TW: Writing – original draft, Writing – review & editing. TL: Writing – original draft, Writing – review & editing.
